# The use of electron microscopy in the diagnosis of focal segmental glomerulosclerosis: are current pathological techniques missing important abnormalities in the glomerular basement membrane?

**DOI:** 10.12688/f1000research.19997.2

**Published:** 2019-11-07

**Authors:** Justin Davis, Alwie Tjipto, Katharine Hegerty, Andrew Mallett

**Affiliations:** 1 Department of Nephrology, Barwon Health, Geelong, Victoria, 3220, Australia; 2 Department of Nephrology, Royal Brisbane and Women's Hospital, Herston, Queensland, Australia

**Keywords:** Collagen IV, Electron Microscopy, Glomerular Basement Membrane, Focal Segmental Glomerulosclerosis, Renal Biopsy.

## Abstract

**Background: **There is an increasing appreciation that variants of the *COL4A* genes may be associated with the development of focal segmental glomerulosclerosis (FSGS). On electron microscopy, such variants may produce characteristic changes within the glomerular basement membrane (GBM). These changes may be missed if glomerular lesions histologically diagnosed as FSGS on light microscopy are not subjected to electron microscopy.

**Methods: **We conducted a retrospective cohort analysis of all patients presenting to two hospitals who received a primary histological diagnosis of FSGS to see if these samples underwent subsequent electron microscopy. Each such sample was also scrutinised for the presence of characteristic changes of an underlying type IV collagen disorder

**Results: **A total of 43 patients were identified. Of these, only 30 underwent electron microscopy. In two samples there were histological changes detected that might have suggested the underlying presence of a type IV collagen disorder. Around one in three biopsy samples that had a histological diagnosis of FSGS were not subjected to electron microscopy.

**Conclusion: **Renal biopsy samples that have a histological diagnosis of primary FSGS not subjected to subsequent electron microscopy may potentially miss ultrastructural changes in the GBM that could signify an underlying type IV collagen disorder as the patient’s underlying disease process. This could potentially affect both them and their families’ investigative and management decisions given potential for implications for transplant, heritability and different disease pathogenesis. This represents a gap in care which should be reflected upon and rectified via iterative standard care and unit-level quality assurance initiatives.

## Abbreviations

Chronic kidney disease (CKD); end stage kidney disease (ESKD); focal segmental glomerulosclerosis (FSGS); glomerular basement membrane (GBM); not otherwise specified (NOS); thin basement membrane nephropathy (TBMN).

## Introduction

Focal segmental glomerulosclerosis (FSGS) is a pathological diagnosis which underpins a variety of progressive renal diseases that commonly result in proteinuria, chronic kidney disease (CKD) and potential end stage kidney disease (ESKD) requiring renal replacement therapy ^1^. FSGS occurs in either a primary. secondary, or genetic form. Primary FSGS is thought to be largely immunological in nature perhaps driven by an elusive permeability factor and secondary FSGS caused by compensatory hyperfiltration due to a previous glomerular injury ^2^. In recent years there has been a greater appreciation of the contribution of underlying genetic causes of this histological pattern. Many of these genetic aetiologies for FSGS have potential clinical significance for at-risk family members, subsequent genetic counselling, future living related kidney transplantation and therapeutics including potential avoidance of immunosuppressive therapies otherwise used for primary immunologically-mediated FSGS. Although initial focus has been on genes that are involved in the maintenance of podocyte structure and function it has become apparent that abnormalities in genes responsible for the structural integrity of the glomerular basement membrane (GBM) may underpin a significant minority of cases of adult-onset FSGS ^3^. Variants in *COL4A3, COL4A4* and *COL4A5* which encode the α3, α4 and α5 chains of type IV collagen respectively, the major constituent of the GBM previously linked to both Alport syndrome and thin basement membrane nephropathy (TBMN) may also underlie cases of FSGS ^4^. This suggestion has biological plausibility. Podocytes, the glomerular epithelial cells responsible for maintenance of the filtration barrier of the glomerulus through which plasma is ultrafiltrated, are not only a source for the α-chains secreted to form the GBM but also are required to be anchored to this structure in order to maintain the aforementioned filtration barrier ^2, 5^. It is not inconceivable then that inherited structural abnormalities of the GBM may lead to subsequent podocyte dysfunction and ultimately the lesion histologically characterised as FSGS. 

The relationship between the three renal conditions intertwined around variants in the *COL4A* genes, Alport syndrome, TBMN, and FSGS is complex and incompletely understood. Whilst previously it was believed that patients heterozygous for variants in *COL4A3* and *COL4A4* would develop TBMN with persistent microscopic haematuria and an otherwise benign prognosis, this traditional thinking has been overturned by the discovery that some pedigrees with these variants will go on to develop significant proteinuria, FSGS lesions and the potential for progressive CKD or ESKD ^6, 7^. More recently, targeted gene sequencing of adults thought to have primary FSGS or steroid resistant nephrotic syndrome (the paediatric equivalent) found that pathogenic or likely pathogenic variants in *COL4A* genes were present in up to 38% of families with familial FSGS, and 3% of those with sporadic FSGS ^3, 8^. These findings highlight the importance of considering variants in these genes in the diagnosis and subsequent management of patients found to have FSGS histologically. 

FSGS is a clinicopathological diagnosis that is made on the review of renal tissue obtained by percutaneous biopsy. This tissue is processed and subjected to several different staining techniques and methods of microscopy, including light, immunofluorescence and electron microscopy ^9^. FSGS, suggested on light microscopy by the finding of focal, segmental lesions with sclerosis, and an increase in the glomerular matrix with obliteration of the capillary lumens only requires this finding in one glomerulus and light microscopy to diagnose ^2^. As such, some specimens may previously have not been sent for further processing and review with other techniques such as electron microscopy even though this histopathology has heterogenous causative aetiologies. The other potential renal lesions that may be caused by *COL4A* gene variants, Alport syndrome and TBMN, cause thinning, lamellation and fraying of the GBM and may also be associated with podocyte foot process effacement, all of which require electron microscopy to visualise ^4^. Similar findings may also be present in FSGS cases associated with *COL4A* variants. One study of Chinese families with familial FSGS noted segmental thinning of the GBM similar to those which may be present with TBMN in four out of five families with a proven *COL4A* variant underlying their familial FSGS (electron microscopy in another family and a sporadic case with proven variants were both noted to be unremarkable) ^10^. At present the sensitivity of these findings remain unknown. It should be noted that for Alport syndrome there are other techniques aside from electron microscopy available for identifying the diagnosis. Immunostaining of a renal biopsy specimen for type IV collagen may demonstrate the absence of alpha 3,4 or 5 chains in up to two thirds of patients, or a skin biopsy may show an absence of an alpha 5 chain ^11, 12^, in addition to the aforementioned genetic testing. Such absence by immunostaining does not appear be present in FSGS ^13^. Given that the pathological diagnosis of FSGS does not routinely require electron microscopy it is therefore conceivable that GBM lesions potentially associated with an underlying type IV collagen variant may have been missed. This represents an opportunity to reflect on our prior clinical behaviour to see if our multidisciplinary diagnostic practice may require improvement. We conducted a retrospective cohort analysis of prior renal biopsy results in two tertiary centres to see how many of those that had been given a histological diagnosis of FSGS were sent for subsequent electron microscopy.

## Methods

### Study background

Our study was a retrospective cohort analysis across two tertiary hospital sites involving the review of prior renal biopsy results that had been given the histological diagnosis of FSGS on light microscopy and how many of these samples subsequently went on to be processed for electron microscopy. In addition, of those samples that were subjected to electron microscopy, we reviewed how many displayed evidence of a potential type IV collagen disorder. This project had ethics approval through Barwon Health’s Research Ethics Governance and Integrity unit and the Royal Brisbane and Women’s Hospital Human Research and Ethics Committee (HREC 18/131 and HREC/18/QRBW/258, respectively). Participants consent was waived by both ethics committees due to the negligible risk nature of data acquisition via retrospective datasets already maintained on electronic health records at both institutions. 

### Eligibility criteria

Patients aged over the age of 18 who received a histological diagnosis of FSGS on native kidney biopsy during the time period of January 1 ^st^ 2008 until July 31 ^st^ 2018 at the first participating hospital and from the 1 ^st^ of October 2013 until the 29 ^th^ of December 2018 at the second were eligible to be included in the retrospective audit. Participants were excluded from the audit if they were less than 18 years of age, underwent kidney transplant biopsy, were defined clinically to have secondary FSGS as opposed to primary disease, received their diagnosis of FSGS or underwent a renal biopsy and pathological review outside of the prespecified time period for each institution. 

### Sample treatment

Samples for biopsy were placed in the following fixatives for processing; formalin for light microscopy, saline soaked gauze with freezing for immunofluorescence, and glutaraldehyde for electron microscopy. Samples for light microscopy are embedded and sectioned at 2- to 3-µm thickness with hematoxylin-eosin, Jones silver, periodic acid-schiff and trichrome staining performed. Immunofluorescence samples are sectioned within a cryostat and placed on prelabelled air-dried slides of the antigen in question. Electron microscopy tissue is processed into plastic, trimmed, cut into a 1-µm section and stained with toludine blue. The images are reviewed in a digital medium ^14^. 

### Identification of participants and data analysis

A search strategy was developed for this retrospective cohort study to identify appropriate patients through local electronic medical record systems using the search term ‘focal segmental glomerulosclerosis’ with the AND operator to combine with ‘glomerulonephritis’ or ‘hereditary nephritis’. These patient results were subsequently reviewed for evidence of a previous percutaneous renal biopsy and report. The data were independently extracted from the included patients by the primary reviewer and collated in a Microsoft Excel document that included information on age, primary diagnosis, included use of electron microscopy and any changes that may be consistent with an underlying collagen disorder including thinning, lamellation and fraying of the GBM. The data extracted was verified by the other three co-authors with all discrepancies resolved through discussion and consensus. 

## Results

### Participant information and diagnoses

From January 2008 through to July 2018 at the first centre and October 2013 until December 2018 at the second, a total of 43 patients were identified as having primary FSGS. The baseline characteristics of the study cohort are provided in Table 1. The median age was 49 years and the patients were predominantly male (55%). The most common underlying histological diagnosis reported in the renal biopsy clinical pathology reports was FSGS not otherwise specified (NOS), followed by familial FSGS with no underlying genetic disorder ascertained. There were small numbers of the cellular, collapsing, perihilar and tip FSGS variants noted. The two most common stages of CKD at the time of presentation were I and III. The results extracted and analyses in this study are available as *Underlying data* ^15^.

**Table 1.** Demographic and clinical characteristics of the patients at baseline.

CHARACTERISTIC	Value, n (%) (unless indicated)
Age, years *	Median (range) 49.95 (23–88)
Sex	
Male	24 (55.81)
Female	19 (44.18)
Underlying diagnosis	
FSGS NOS	31 (72.09)
FSGS cellular variant	2 (4.65)
FSGS collapsing variant	1 (2.32)
FSGS Familial	3 (6.97)
FSGS Perihilar	3 (6.97)
FSGS Tip	3 (6.97)
Electron microscopy report available for biopsy?	
Yes	30 (69.76)
No	13 (30.23)
Collagen disorder suggested	2 (2.81)
Stage at diagnosis	
I	12
II	9
III	12
IV	8
V	2
Unknown	0

*Values given as median (range).

### Use of electron microscopy

Of the 43 patients identified, samples from 30 underwent electron microscopy after initial light microscopy and immunofluorescence. Two of these samples showed signs on electron microscopy that might be consistent with an underlying type IV collagen glomerular basement membrane disorder. Microscopic haematuria was not noted in either of these two patients. Of the 13 samples that did not undergo electron microscopy, four had no glomeruli present within the processed core. The remaining nine samples had no documented reason.

**Figure 1.**
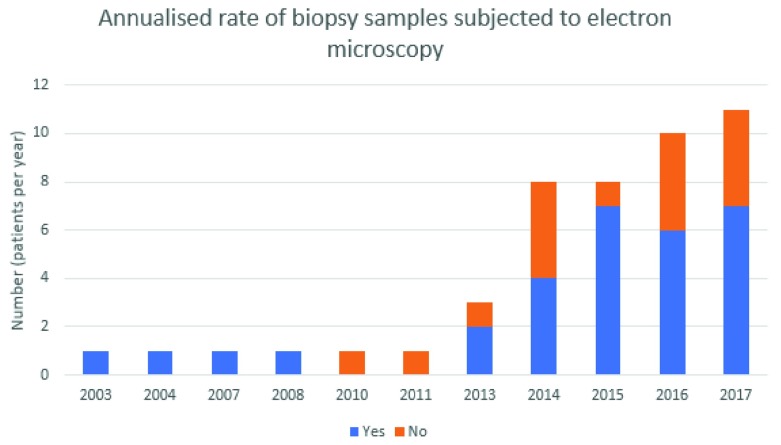
Annualised rate of biopsy samples subjected to electron microscopy.

The overall percentage of biopsy samples for which data were available that were not subjected to electron microscopy was 30%, of which close to 21% had no documented reason for not undergoing electron microscopy. The annualised rate of biopsy samples that were not subjected to electron microscopy varied with time ( Figure 1). The number of biopsy reports available for analysis prior to 2013 was only one per year at most; however, from 2013 onwards the rate progressively increased to between 3 and 11 cases of FSGS diagnosed at histopathology between the two institutions per year. The annualised rate of electron microscopy from 2013 onwards varied each year, ranging from 50 to 87%. Overall 30% of biopsy samples did not receive electron microscopy. The overall rate of electron microscopy did not significantly change across the study period between the two centres.

## Discussion

This retrospective cohort analysis demonstrated that about two-thirds of native kidney biopsy samples across two institutions that were deemed to have primary FSGS underwent subsequent electron microscopy. Of those that did, two were reported to have characteristics that might be consistent with an underlying type IV collagen disorder. In both samples, electron microscopy revealed a diffusely thin GBM, with the second additionally identifying early focal splitting of the GBM. The first sample, in which the patient had the tip variant of FSGS, was suggested to be consistent with TBMN whereas there was no pathological comment made about the second from a patient with FSGS NOS. FSGS (NOS) was the most common lesion described in this study, consistent with prior reports ^2^. Notably, close to one in three cases of primary FSGS were not proceeding to electron microscopy despite an indication to do so and 1 in 20 cases within our cohort had structural changes that were consistent with an underlying type IV collagen variant. Whilst some samples were unable to undergo electron microscopy due to a lack of glomeruli in the biopsy core, in the majority of the others it is unclear why subsequent electron microscopy did not occur. The annualised rate of biopsy samples not subjected to electron microscopy varied by year, but on average around one in three samples were not subjected to electron microscopy despite receiving a histological diagnosis of FSGS. 

Curiously, neither of the two patients in whom electron microscopy was suggestive of an underlying type IV collagen disorder was noted to have haematuria on their urinalysis at the time of presentation. Both of these samples were noted to have a thin basement membrane on electron microscopy, with focal splitting noted in one with an average thickness of 230.72 nm given. This is in keeping with a study of Chinese FSGS families in which four patients with a demonstrated mutation in *COL4A* and familial FSGS all had findings suggestive of TBMN on electron microscopy ^10^. Unfortunately due to the retrospective nature of this study we were unable to send any samples for immunostaining of type IV collagen. Regrettably he authors also do not have any data as to whether the two patients in whom electron microscopy was suggestive of a type IV collagen disorder proceeded to subsequent genetic testing for *COL4A* mutations or whether they were subjected to immunosuppression and their subsequent clinical course. The authors suggest that sequencing of these electron microscopy variants when found could be an extension of current research to expand our knowledge in this field.

**Figure 2.**
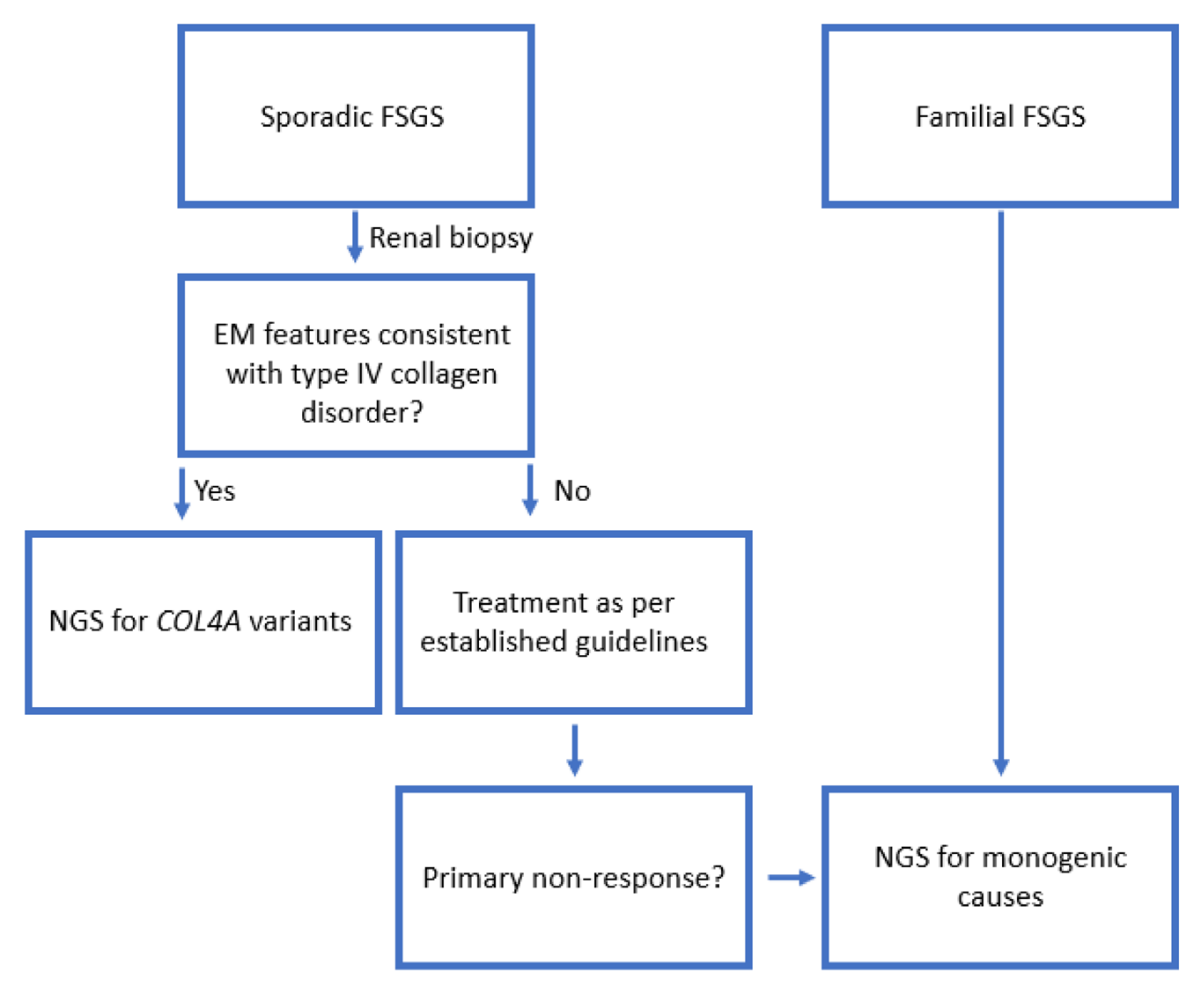
Proposed workflow for investigation of patients in this demographic. There is an increasing appreciation that some adult patients with otherwise apparently sporadic FSGS with a primary non-response to immunosuppressive therapy will harbour a genetic form of FSGS, which should be investigated appropriately. We propose that any patient with electron microscopy features suggestive of a type IV collagen variant proceed to genetic testing for confirmation. Familial cases should proceed to sequencing ^8, 17, 18^.

There is an increasing body of evidence indicating that inheritable variants in *COL4A* may underlie a proportion of cases of FSGS, with up to 12.5% cases of autosomal dominant FSGS attributable to *COL4A3* in some cohorts ^10^. Not subjecting these renal biopsy samples to electron microscopy represents a potential gap in the investigation and subsequent management of such patients given they are much less likely to respond to immunosuppressive therapy ^16^ which has otherwise been classically indicated ( Figure 2).

This study was designed as a retrospective cohort study looking at the number of samples sent for electron microscopy, as well as any potential changes which might be consistent with a type IV collagen glomerular basement membrane disorder. It is important to recognise that not all groups have found the characteristic changes associated with the type IV collagen disorders such as Alport’s Syndrome or TBMN on electron microscopy. One study described the typical pathological changes of FSGS but not the glomerular basement membrane abnormalities characterising Alport syndrome or TBMN in patients known to have variants in either *COL4A3* or *COL4A4* ^7^. It is thus possible that a lack of classical findings for a type IV collagen glomerular basement membrane disorder may have accounted for the low number of those with GBM features on electron microscopy consistent with a type IV collagen disorder noted within our study. Indicating against this, however, another study suggested that biopsy samples from patients with the classical features of Alport Syndrome or TBMN showed podocyte detachment which might be expected and subsequently cause FSGS-type lesions ^4^. Other studies which have looked at electron microscopy in FSGS cases have similarly found low numbers of abnormalities that may be consistent with an underlying collagen 4 disorder ^3, 8^, which suggests the overall number of abnormalities to be found via electron microscopy may be low.

The process by which variants within the *COL4A* genes might cause FSGS remains unclear, particularly given their clear association with Alport Syndrome and TBMN. One proposal is that the ultrastructural changes induced by the type IV collagen variants, perhaps under the influence of modifier genes such as laminin, result in impaired podocyte attachment to the glomerular basement membrane which leads to accelerated podocyte detachment, subsequent foot process effacement as a response to the increased shear stress induced by the denuded basement membrane and at a critical level of podocyte loss collapse of the capillary network with the appearance of the classical segmental sclerotic lesion ^2, 4, 19^. It also remains unclear as to whether the changes of FSGS are a secondary process occurring in those with TBMN or whether the type IV collagen variants are capable of causing primary FSGS ^7, 20^. FSGS occurring as a secondary process to other basement membrane abnormalities may explain why immunosuppressive therapy has traditionally been less effective in inherited forms of FSGS, although there are case reports of the successful use of the calcineurin inhibitor cyclosporine for some patients harbouring inheritable type IV collagen disorders ^6^.

In summary, this study has found that not all biopsy samples that had primary FSGS as a histological diagnosis were subjected to subsequent electron microscopy. This may have potentially led to inadvertently overlooking characteristic basement membrane abnormalities, which may suggest an underlying and heritable type IV collagen disorder. These findings reflect an opportunity to change practice in order to better investigate, counsel and provide clinical management to these and future patients.

## Data availability

### Underlying data

Figshare: Davis *et al*. FSGS biopsy audit.xlsx. https://doi.org/10.6084/m9.figshare.8949032.v1 ^15^.

This project contains the variables extracted for each individual retrospectively assessed in this study.

Data are available under the terms of the Creative Commons Attribution 4.0 International license (CC-BY 4.0).
