# Supplementary material for: The use of electron microscopy in the diagnosis of focal segmental glomerulosclerosis: are current pathological techniques missing important abnormalities in the glomerular basement membrane?
Source: F1000Res. 2019 Nov 7;8:1204. Originally published 2019 Jul 26. [Version 2] doi: 10.12688/f1000research.19997.2 (PMC6854873; doi:10.12688/f1000research.19997.2)
Supplement: Peer review history

# Peer review: Reviewer response for version 2

**DOI:** 10.5256/f1000research.23359.r56307

**Published:** 2019-11-13

**Authors:** Rachel Lennon, James McCaffrey

**Affiliations:** 1 Wellcome Trust Centre for Cell-Matrix Research, Faculty of Biology, Medicine and Health, University of Manchester, Manchester, UK; 2 Department of Histopathology, Cambridge University Hospitals NHS Foundation Trust, Cambridge, UK

In this revised version of the manuscript we are satisfied that the authors have made improvements where these were possible. In particular data regarding the specificity of GBM changes found by EM for identifying familial FSGS (4/5 families with a proven COL4A variant and FSGS had GBM thinning). We accept that sensitivity of EM abnormalities is difficult to ascertain.

 It is unfortunate that the patients with GBM abnormalities identified in the present study have been lost to follow up. However, we thank the authors for making efforts to retrieve clinical information, which may have strengthened the argument that the patients did harbour a COL4A variant.

 The multi-modality approach to identifying patients with an underlying genetic cause for FSGS is more clearly emphasised. We agree with the workflow shown in Figure 2 that if GBM structural abnormalities are identified by EM in cases of sporadic FSGS, patients should be thoroughly investigated for a underlying genetic cause by NGS. This may also be relevant in familial cases where GBM abnormalities are identified by electron microscopy.

 The title of the article now more accurately reflects the data provided.

 We are happy to approve this article without further revisions.

We confirm that we have read this submission and believe that we have an appropriate level of expertise to confirm that it is of an acceptable scientific standard.

# Peer review: Reviewer response for version 1

**DOI:** 10.5256/f1000research.21949.r54539

**Published:** 2019-10-28

**Authors:** Constantinos Deltas

**Affiliations:** 1 Molecular Medicine Research Center, Department of Biological Sciences, University of Cyprus, Nicosia, Cyprus

This retrospective study focuses on the fact that renal biopsies are not always evaluated by electron microscopy (EM). The authors found 43 renal biopsies in the records of two hospitals, where the diagnosis of FSGS was made, for which EM had been performed only in 30. Most importantly, in two of the biopsies studied by EM, there was evidence for a collagen IV disorder but had not been followed up. This has important implications in view of numerous reports in the past decade and more, according to which the histological diagnosis of FSGS could be on the background of collagen IV mutations, which cause Alport syndrome or thin basement membrane nephropathy.

 I agree with all points raised by the previous reviewer and which I do not intend to repeat here. I would only emphasize two things:

1. I believe there should be no renal biopsy attempted if it is not going to be studied fully by light microscopy, immunofluorescence studies and EM. It is an invasive procedure accompanied by some risk and when performed it must be fully exploited as very significant results may come up, which bear implications on treatment, inheritance patterns and family planning.
2. The authors found two patients who had undergone EM and there were findings indicative of a collagen IV disorder. They should have proceeded to genetic testing to verify this and strengthen their case. Even belatedly, this could be of benefit not only to the patients but also to family members. 

I confirm that I have read this submission and believe that I have an appropriate level of expertise to confirm that it is of an acceptable scientific standard.

# Response

**Published:** 2019-11-06

**Authors:** Justin Davis

**Affiliations:** St Vincent's Hospital, Sydney, Australia

To the second reviewer who kindly reviewed our article;

On behalf of my co-authors, I wish to thank you for taking the time out of your busy work schedule to read through our manuscript and make suggestions which only serve to strengthen the article overall and make it a better piece for submission. We would like to ensure we have addressed both concerns pointed out below.

1. The authors agree with this point and feel most (if not all, at least in the case of native kidney) biopsies should be subjected to appropriate microscopic techniques which includes electron microscopy. It is unclear why some of our samples unfortunately were not, and remains a clear source of potential quality improvement that can be fed back, particularly when suggestive abnormalities may be found that influence further management. 

2. The authors too are curious as to the genetic status of the two individuals who had suggestive EM changes noted within the body of the manuscript. Unfortunately on perusal of the data available they have been lost to follow up without a clear genetic diagnosis at this time.

Once again, many thanks for taking the time to review our work

# Peer review: Reviewer response for version 1

**DOI:** 10.5256/f1000research.21949.r53353

**Published:** 2019-09-18

**Authors:** Rachel Lennon, James McCaffrey

**Affiliations:** 1 Department of Histopathology, Cambridge University Hospitals NHS Foundation Trust, Cambridge, UK; 2 Wellcome Trust Centre for Cell-Matrix Research, Faculty of Biology, Medicine and Health, University of Manchester, Manchester, UK

This is a retrospective study of kidney biopsies from 43 patients over a 10-year interval. The authors retrieved native biopsies from patients with a primary histological diagnosis of FSGS in two tertiary hospitals. They determined the number of biopsies that had both histological evidence of FSGS and accompanying ultrastructural analysis with electron microscopy (EM). 13/43 biopsies did not have evaluation by EM. In 2 biopsies that had both histology and EM, abnormalities of the glomerular basement membrane (GBM) were identified. The authors advocate an increase in the use of EM with a diagnosis of FSGS to identify a subset of patients with an underlying genetic disorder affecting type IV collagen.

 Overall the study is a simple, retrospective review design and it is well presented. It highlights the important observation and increasing recognition that pathogenic variants in *COL4A *genes are present in a high proportion of families with familial FSGS and also in individuals with sporadic FSGS. There is a pressing clinical need to identify these patients and, as such, this study comes at an opportune time. We have the following questions and suggestions for improvement:

  

1. The authors could present a stronger argument for EM being the investigation of choice for identifying patients with *COL4A *mutations. Although the authors discuss the variable EM findings in patients with Alport syndrome or TBMN, this could be expanded. How sensitive are the GBM findings for *COL4 *mutations? Not all patients with *COL4A *variants have typical EM findings, and furthermore suggestive GBM abnormalities may be found in other genetic disorders including mutations in *MYH9 *and *LMX1B*.
2. There would be additional value if the authors could propose a pros/cons comparison of EM versus genetic screening with for example an NGS gene panel to identify these patients. This could be presented as a proposed workflow for investigation. 
3. The authors identify 2 patients with EM findings suggestive of a *COL4A *disorder. However, there are no data detailing whether these patients did in fact harbour a *COL4A *variant (notably neither of them had haematuria at presentation). Are any data available, for example, on clinical response to immunosuppression, or any sequencing data?  

 

**Minor comments**

  

1. The title described the study as an 'audit' but the text repeatedly refers to a 'retrospective cohort analysis.' As there are no defined standards against which clinical practice is being measured here, perhaps remove 'audit' from the title? 
2. The Y-axis in Figure 1 should be labelled.
3. Which FSGS variant did the 2 patients with EM abnormalities fall into?  
4. Improve consistency with nomenclature—use of conventional gene ( *COL4A*) or protein names (type IV collagen).

We confirm that we have read this submission and believe that we have an appropriate level of expertise to confirm that it is of an acceptable scientific standard, however we have significant reservations, as outlined above.

# Response

**Published:** 2019-11-06

**Authors:** Justin Davis

**Affiliations:** St Vincent's Hospital, Sydney, Australia

To the first reviewer who kindly reviewed our article;

 On behalf of my co-authors, I wish to thank you for taking the time out of your busy work schedule to read through our manuscript and make suggestions which only serve to strengthen the article overall and make it a better piece for submission. We would like to respond to each of the suggestions listed below.

 Major changes include:

 1. The authors agree that the extrapolation of EM changes in COL4A variants could be further elaborated on and have introduced a new section in the introduction looking at a Chinese cohort with both confirmed EM and COL4A mutations. EM is unlikely to be the preferred investigation for such cases, but if suggestive abnormalities are found by happenstance then investigation for a potential COL4A variant seems appropriate based on our current understanding. Unfortunately, due to small sample sizes, the sensitivity of such findings cannot be commented on at this time and forms a body of ongoing research into these potential links.

 2. The authors have included a new figure (Figure 2) with a proposed worksheet for investigation into patients whom may present in this manner.

 3. Unfortunately, the patients whom had EM abnormalities suggestive of a type IV collagen disorder have been lost to follow up, and the data on them is simply not able to extrapolated for this particular article (a shame as the authors were curious about such things too).

 Minor changes include:

 1. The title has been reworded to avoid confusion, and text within the paragraphs altered to reflect this change away from audit.

 2. The Y axis in figure 1 is now labelled appropriately.

 3. Tip and NOS, now specified within the manuscripts main paragraphs.

 4. The authors thank the reviewers for pointing out small errors of inconsistency which can creep into a document like this, and have amended them appropriately

 Again, many thanks for taking the time out to read through this manuscript.
